# Viral Capsid Change upon Encapsulation of Double-Stranded DNA into an Infectious Hypodermal and Hematopoietic Necrosis Virus-like Particle

**DOI:** 10.3390/v15010110

**Published:** 2022-12-30

**Authors:** Wattana Weerachatyanukul, Pauline Kiatmetha, Ponlawoot Raksat, Supawich Boonkua, Orawan Thongsum, Pitchanee Jariyapong, Charoonroj Chotwiwatthanakun, Puey Ounjai, Zoltan Metlagel

**Affiliations:** 1 Department of Anatomy, Faculty of Science, Mahidol University, Rama VI Rd., Ratchathewi, Bangkok 10400, Thailand; 2 Department of Biology, Faculty of Science, Mahidol University, Ratchathewi, Bangkok 10400, Thailand; 3 Department of Medical Science, School of Medicine, Walailak University, Thasala District, Nakhonsrithammarat 80160, Thailand; 4 Academic and Curriculum Division, Nakhonsawan Campus, Mahidol University, Nakhonsawan 60130, Thailand; 5 Lawrence Berkeley National Laboratory, Molecular Biophysics and Integrated Bioimaging Division, University of California, Berkeley, CA 94720, USA

**Keywords:** IHHNV, virus-like particles, dsDNA, encapsulation, capsid maturation

## Abstract

In this study, we aimed to encapsulate the sizable double-stranded DNA (dsDNA, 3.9 kbp) into a small-sized infectious hypodermal and hematopoietic necrosis virus-like particle (IHHNV-VLP; T = 1) and compared the changes in capsid structure between dsDNA-filled VLP and empty VLP. Based on our encapsulation protocol, IHHNV-VLP was able to load dsDNA at an efficiency of 30–40% (*w*/*w*) into its cavity. Structural analysis revealed two subclasses of IHHNV-VLP, so-called empty and dsDNA-filled VLPs. The three-dimensional (3D) structure of the empty VLP produced in *E. coli* was similar to that of the empty IHHNV-VLP produced in *Sf9* insect cells. The size of the dsDNA-filled VLP was slightly bigger (50 Å) than its empty VLP counterpart; however, the capsid structure was drastically altered. The capsid was about 1.5-fold thicker due to the thickening of the capsid interior, presumably from DNA–capsid interaction evident from capsid protrusions or nodules on the interior surface. In addition, the morphological changes of the capsid exterior were particularly observed in the vicinity of the five-fold axes, where the counter-clockwise twisting of the “tripod” structure at the vertex of the five-fold channel was evident, resulting in a widening of the channel’s opening. Whether these capsid changes are similar to virion capsid maturation in the host cells remains to be investigated. Nevertheless, the ability of IHHNV-VLP to encapsulate the sizable dsDNA has opened up the opportunity to package a dsDNA vector that can insert exogenous genes and target susceptible shrimp cells in order to halt viral infection.

## 1. Introduction

Infectious hypodermal and hematopoietic necrosis virus (IHHNV), or *Penaeus stylirostris* densovirus (*Pst*DNV), is a non-enveloped virus in the *Densovirinae* subfamily of the *Parvovirinae* family, which specifically infects invertebrates and causes mass mortality in shrimps [1]. A near-atomic, three-dimensional (3D) structure of the empty IHHNV-VLP at 8 Å has been revealed [2]. It consists of eight strands of anti-parallel β-barrel jelly roll structure having icosahedral symmetry axes similar to those reported for other parvoviruses. The capsid structure of this smallest-sized parvovirus shows many unique characteristics compared to those of other invertebrate densoviruses or vertebrate parvoviruses. Most interestingly, IHHNV-VLP contains only a single type of capsid protein (MW = 37.5-kDa) of 60 subunits arranged into a 28 nm icosahedral particle (T = 1), whereas others comprise many subtypes of capsid protein. In addition, the conserved domain of phospholipase A2 (PLA2) appears to be reminiscent of IHHNV capsid organization, suggesting minimal PLA2 requirement for host cell infection by IHHNV [2]. Capsid integrity is maintained by coordinated divalent metal ions between neighboring five-fold-symmetry-related subunits rather than intra- or inter-molecular disulfide bonds [2].

Knowledge of the viral capsid structure is crucial not only for studying viral life cycles but also for designing antiviral compounds for viral infection management. The conformational change in the viral capsid that transits it from a precursor particle to a mature virion within the host cell has been reported for many viruses [3,4,5,6]. This structural alteration, known as capsid maturation, is processed or triggered initially either by activation of viral protease [7] or by the onset of genome packaging followed by a programmed capsid shell assembly in a cooperative, irreversible, and transformational manner [8]. In the case of human papillomavirus, shrinkage of the capsid surface was found during capsid maturation [9]. In rhinovirus, the channel on the five-fold axis is opened through a considerable twisting of the fivefold surrounding units to allow the passage of RNA [10]. Although cryoEM information on the empty viral capsid (or VLP) structures in shrimp non-enveloped viruses, including *Macrobrachium rosenbergii* nodavirus (at 7 Å) [11] and IHHNV (8 Å) [2], is available, little is known about whether the capsids of these viruses undergo any changes to facilitate genome packaging, either during VLP encapsulation or the viral maturation process. Here, using a dsDNA designed from the IHHNV genome, we aimed to encapsulate the sizable dsDNA vector into IHHNV-VLP and further observe the change in capsid structures in comparison with the empty IHHNV-VLPs. In addition, it is expected that encapsulation of dsDNA could be a vector for inserting exogenous genes into IHHNV-VLP and targeting susceptible shrimp cells, which could be a particularly useful strategy for antiviral application.

## 2. Materials and Methods

### 2.1. Recombinant IHHNV Capsid Protein Production and Purification

Amplification of the IHHNV capsid gene followed our recently described method [12]. In brief, the PCR reaction was conducted using an ORF3 sequence of IHHNV as a template and a set of primers (F: 5′CATATGTGCG CCGA TTCAACAAGAGCAA3′ and R: 5′CTCGAGTTAATGATGATGATGATGATGGTTAGTATGCATAACATAACATTTG3′). The PCR conditions, product ligation into a pGEM-T Easy cloning vector (Promega, Madison, WI, USA) and the transformation of *Escherichia coli* (*E. coli*), followed our published methods [12]. The positive clones were screened, and the IHHNV capsid gene was inserted into a pET-17b expression vector (Novagen, Darmstadt, Germany), which was later transformed into *E. coli* (Rosetta strain) for protein expression. The bacteria were cultured in LB broth containing 50 µg/mL of ampicillin at 25 °C until the absorbance reached 0.6–0.8 at 600 nm (A600). After induction by 1 mM isopropyl β-d-1-thiogalactopyranoside (IPTG), the cells were pelleted by centrifugation (5400× *g*, 4 °C, 10 min), and the pellet was re-suspended in PBS containing 500 mM NaCl and 2 mM phenylmethylsulfonyl fluoride (PMSF). The cells were ruptured by sonication (100 Hz, 20 sec, 10 cycles) and centrifuged (12,000× *g*, 10 min), and the supernatant was subjected to purification through nickel column chromatography (Qaigen, Hilden, Germany) using an imidazole-based buffer (250 mM imidazole, 500 mM NaCl, pH 7.4). For cryoTEM imaging, the proteins were further purified by 20–50% sucrose discontinuous gradient centrifugation and dialysis. The patterns of all protein fractions during the purification steps were resolved by 12.5% SDS-PAGE and subjected to Coomassie blue staining.

### 2.2. Encapsulation of Double-Stranded DNA into VLP Cavity

Full-length dsDNA, designed from the IHHNV genome (3.9 kbp, accession # AF218166.2), was commercially synthesized (Gene Universal Inc., Newark, DE, USA) with subsequent sequence verification. Encapsulation of this sizable dsDNA followed the encapsulation protocol reported earlier [13]. Approximately 5 µg of IHHNV-VLP was disassembled in the disassembling buffer containing 50 mM Tris-HCl (pH 7.5) with 1 mM ethylene glycol tetraacetic acid (EGTA), 20 mM dithiothreitol (DTT), and 150 mM NaCl, for 1 h at room temperature. An equal amount of 3.9 kbp dsDNA was added into the suspension and allowed to stand for 1 h at 4 °C. Thereafter, the icosahedral structure of VLP was reformed by adding the reassembling buffer (50 mM CaCl_2_ in Tris-buffered saline, TBS) into the VLP suspension, to make a final concentration of 5 mM CaCl_2_, and allowed to stand for 1 h. The VLPs were then either treated (15 and 30 min) or non-treated (0 min) with 0.2 U DNase-I to digest any loosely bound DNA on the VLP surface. The VLPs were collected by ultracentrifugation at 246,000× *g* (4 °C, 2 h), and the VLP pellet was resuspended in TBS. The efficiency of dsDNA encapsulation was checked by 1% agarose gel electrophoresis followed by ethidium bromide staining.

### 2.3. Cryo-Transmission Electron Microscopy

IHHNV-VLPs that underwent the disassembling/reassembling process to encapsulate dsDNA were used in this study. After encapsulation, the samples were deposited on glowed-discharged 200-mesh C-flat grids (EMS, Hatfield, PA, USA). The grids were then rapidly vitrified in liquid ethane using a Vitrobot Mark IV (FEI-ThermoFisher Scientifics, Hillsboro, OR, USA). The samples were visualized on a JEOL3100 TEM microscope operated at 300 kV and equipped with a K2 Summit detector (Gatan, Pleasanton, CA, USA). They were observed at a magnification of 45,000× with a pixel size of 1.2 Å and a defocus range of 1.0 to 1.5 µm. The other TEM imaging parameters included: total electron dose = 27.5 electrons/Å^2^, exposure time = 10 s, frame rate = 400 frames/s, Cs = 3.4 mm, tilt = 0°, dimension of micrographs = 3710 × 3838 nm, number of frames = 20, number of micrographs = 220, and number of particles extracted = 125 particles/micrograph. Two types of VLP (empty and dsDNA-filled, refer to Figure 2D for its classification) were selected for analysis and image reconstruction.

### 2.4. Three-Dimensional Image Reconstruction and Image Analysis

The particles were boxed at the size of 384 pixels from micrographs without astigmatism, drift, or charging effect and used for image processing by EMAN and CisTEM1 software [14]. The density map was initially constructed from approximately 1200 boxed particles with a resolution of approximately 16 Å. To refine the maps, amplitude correction was applied while the new particles were added to improve the map resolution using the default mode of 3D refinement in EMAN2 software. In addition, the icosahedral symmetry was also imposed onto the reconstructed maps to aid the refinement process. The final density maps were reconstructed from 9394 (empty) and 3515 (dsDNA-filled) boxed particles to achieve their final resolution, which was determined by a cutoff value of Fourier shell correlation (FSC). The generated 3D maps were operated and visualized by UCSF Chimera software.

## 3. Results

### 3.1. IHHNV-VLP Could Encapsulate Double-Stranded DNA

**Figure 1:**
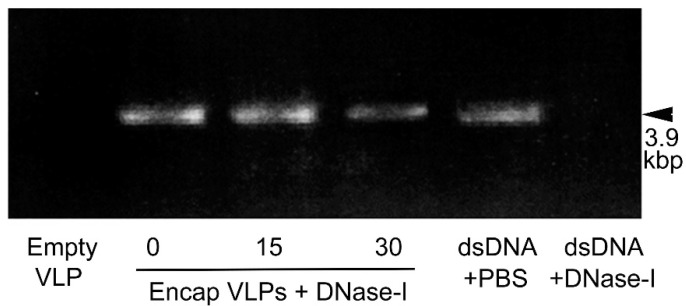
Agarose gel electrophoresis (1%) of dsDNA encapsulated in VLP using disassembling/reassembling method (see Materials and Methods for details). The presence of DNA in the VLP cavity was proved by DNase-I digestion at different times (Lanes 2, 3, and 4). Lane 1 = VLP alone, Lanes 5 and 6 = 5 µg free dsDNA without and with DNase-I, respectively.

Although the ability of IHHNV-VLP to encapsulate smaller amounts of dsDNA has been demonstrated [12], the IHHNV-VLP is quite small, with a triangulation number of 1 (T = 1) and a size of about 28 nm; therefore, its ability to encapsulate dsDNA designed from the full length of the IHHNV genome (3.9 kbp), was initially tested. The structure of the capsid was also examined by cryoTEM. In this study, we produced our recombinant IHHNV-VLP in *E. coli* (Rosetta) since it is practically easier and faster than other reported systems. In addition, its controllable ability to undergo disassembly/reassembly for cargo encapsulation has been well-recognized [12]. Recombinant IHHNV capsid protein produced in *E. coli* could be purified as a single-banded protein at 37.5 kDa (Supplementary Figure S1) with a purity of >95%, as reported earlier [12]. Through our modified disassembling/reassembling process, approximately 50% of the final amount of dsDNA (about 2.5 µg out of 5 µg as determined by densitometric analysis of the 3.9 kbp DNA band) was packed into or associated with VLPs. It should be noted that treatment of the encapsulated VLP with DNase-I resulted in the reduction of DNA band density (Figure 1, lanes 2 and 4), suggesting the conjugation of dsDNA onto the outer surface of VLP. However, we were also aware that the metastability of capsid structure, such as that shown in cowpea chlorotic mottle virus [15], may play a part in the partial dsDNA digestion in this study. The control, in which free dsDNA was exposed to 0.2 U DNase for 30 min, showed a complete diminishing of DNA band intensity (lane 6), while those exposed to PBS showed minimal change in the band intensity (lane 5). The results thus indicate the ability of IHHNV-VLP to load a sizable dsDNA in similar quantities to those of dsRNA or DNA plasmid that have been shown earlier [12,16].

### 3.2. IHHNV-VLP Particle Classification and Its Structural Comparison with the Empty IHHNV-VLP Produced in Sf9 Insect Cells

**Figure 2:**
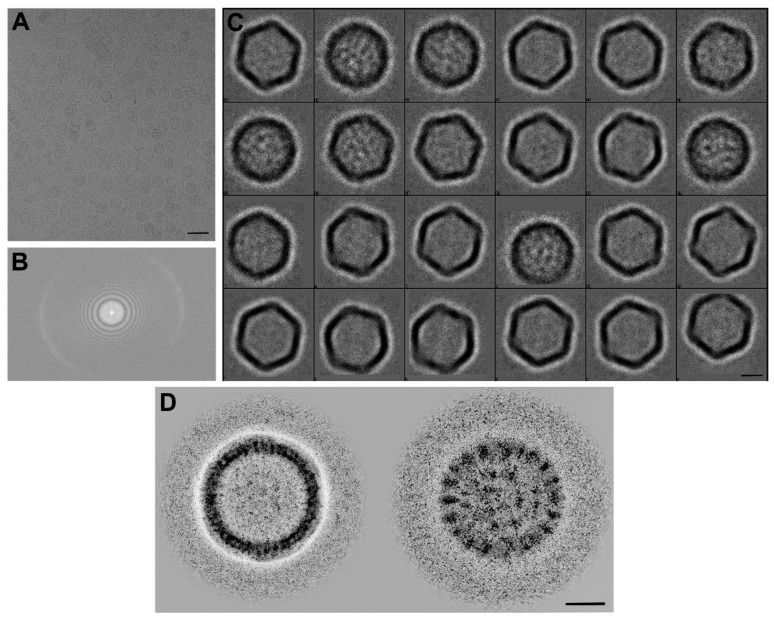
Representative cryoTEM image (**A**), its power spectrum (**B**), and the selected particles (**C**) for class averaging by EMAN2 (2D analysis) and CisTEM1 software. Panel (**D**) demonstrates two subclasses of particles (shown by the back-projections of reconstructions), including empty and genome-filled particles, which showed different electron densities in the VLP cavity. Bars in (**A**) = 50 nm, (**C**,**D**) = 10 nm.

Upon loading of dsDNA into the VLP cavity, approximately 220 micrographs of these particles were acquired by cryoTEM. In the representative cryoTEM images, there were several types of particles present in the individual micrograph, including opened particles, misassembled particles, and half-loaded particles with intermediate electron density (Figure 2A). The power spectrum during TEM image acquisition and the representative boxed particles are shown in Figure 2B,C. Two classes of the particles (shown as a back projection of the reconstruction, Figure 2D) were selected for analysis, one of which was typically the “empty” type (having no dsDNA material inside, left panel), with a hexagonal shape, and the other was a “filled” type with the electron opaque material (presumably encapsulated dsDNA, right panel). The numbers of selected particles were 9394 (for empty) and 3515 (for genome-filled), and resolutions of 10.2 Å (empty) and 13.1 Å (dsDNA-filled) were obtained at the Fourier shell correlation (FSC) cut-off value of 0.143 (Supplementary Figure S2).

**Figure 3:**
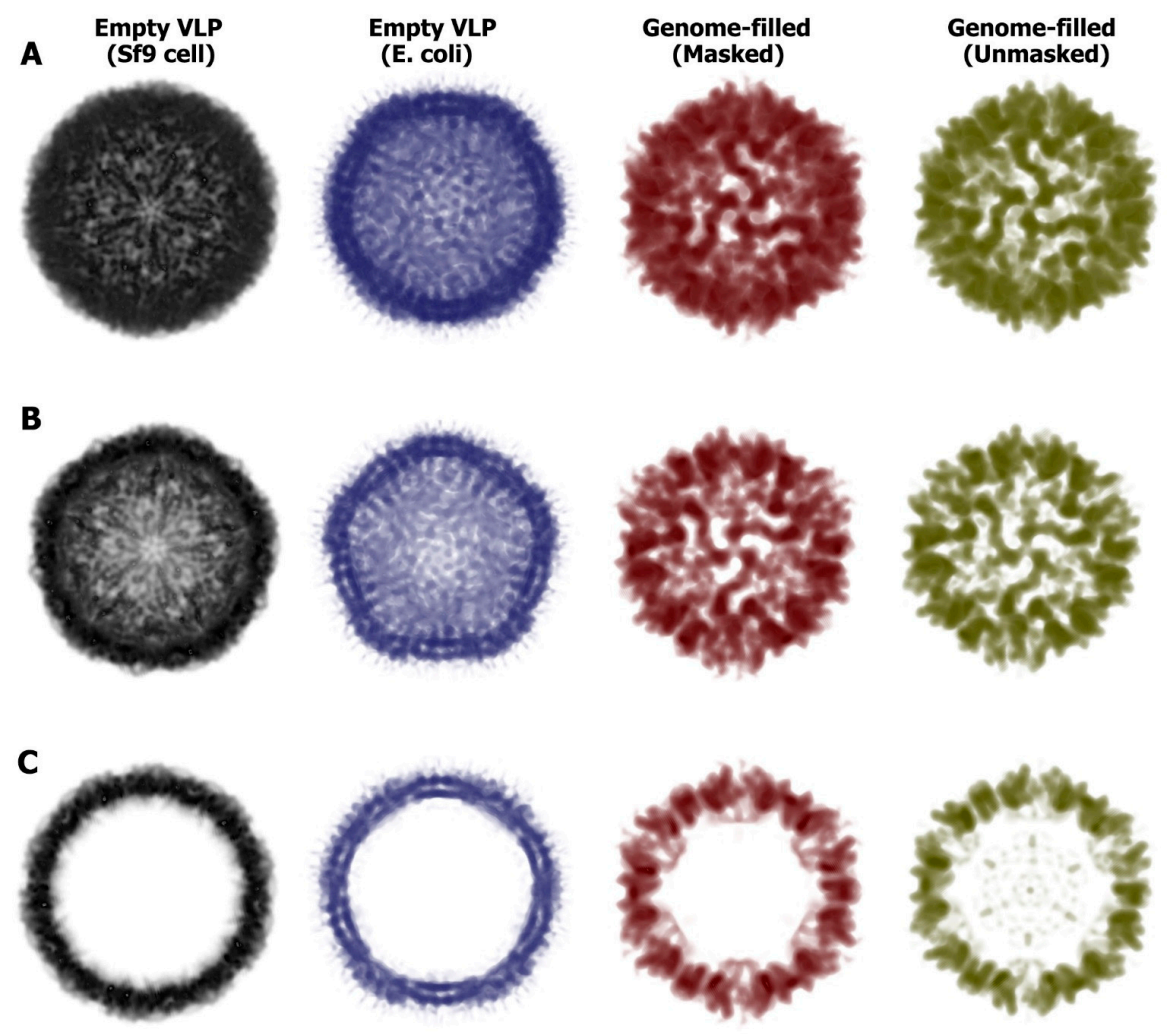
Electron density maps of the empty and dsDNA-filled VLPs. The data from empty IHHNV-VLP (PDB accession # 3N7X) produced in *Sf9* insect cells were reconstructed (first column, solid dark) for the purpose of comparison with the empty IHHNV-VLP produced in *E. coli* (second column, purple) in the full particle view (row (**A**)), half-sphere view (row (**B**)), and mid-section view (row (**C**)). The maps of dsDNA-filled VLP are shown as the genome-masked (third column, deep red) and unmasked (fourth column, green) particles in views corresponding to the empty VLP.

Since our recombinant IHHNV-VLP was produced in *E. coli*, we initially compared the features of our reconstructed empty VLP with that produced in the cultured *Sf9* cells (PBD accession # 3N7X) reported earlier [2]. Overall, the general features of the IHHNV-VLP produced in either bacteria or insect cells were similar in their gross structures but rather different in their fine structures and subunit organizations. The outermost surface diameters of the particles produced by *E. coli* were about 255 Å, whereas those of *Sf9* were 270 Å. The most predominant protruding domains were located at the five-fold axes, whereas the surface depression (canyon) could be observed at two-fold axes in both particles. However, the five-fold and two-fold axes of the VLP produced in *Sf9* showed more well-defined protrusion and delineated-canyon structures, respectively, than those of the *E. coli* produced particles (Figure 3, first and second columns).

### 3.3. Structural Comparison between Empty and dsDNA Encapsulated IHHNV-VLPs

**Figure 4:**
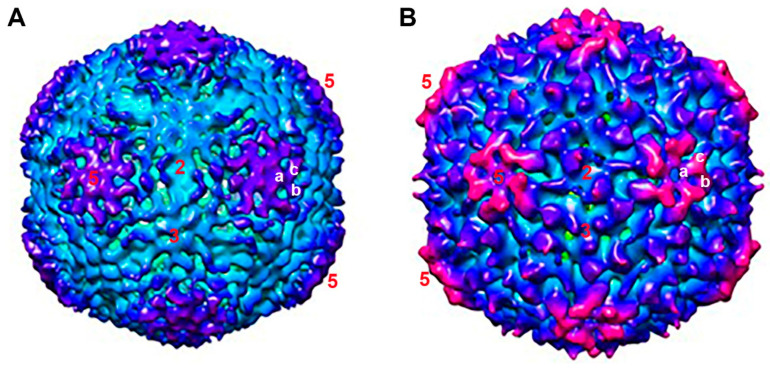
Comparative iso-surface maps of the empty VLP (**A**) and dsDNA-filled VLP (**B**). The color codes in the maps represent vertical distance from the center towards the periphery: yellow, 0–100 Å; green, 100–150 Å; cyan, 150–200 Å; deep blue, 200–250 Å; and pink, 250–300 Å. Numeric labels on the VLP represent two-, three-, and five-fold axes on the surface of IHHNV-VLP. Letters a-c are tripod feature around each five-fold axis.

**Figure 5:**
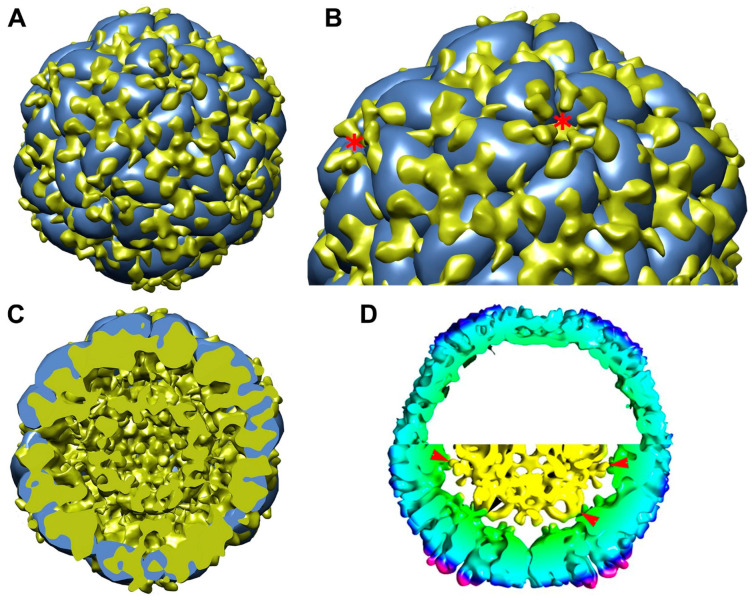
Superimposition of the iso-surface maps (**A**,**B**), half particle view (**C**), and the comparative mid-sections between the empty and genome-filled IHHNV-VLPs (**D**). Note that most capsid changes were observed in the vicinity of five-fold axes exteriorly (asterisks) and the protruding “nodular” structures interiorly (arrowheads), presumably representing the interaction points between the encapsulated genome and capsid, which would likely lead to inward thickening of capsid structure.

We further compared capsid structural alteration between the empty and dsDNA-filled IHHNV-VLPs. Generally, the size of the dsDNA-filled VLP (269 Å) was slightly bigger than that of the empty VLP (255 Å). However, drastic alterations in the capsid structures of dsDNA-filled compared to empty VLPs were clearly noted—the thickest capsid was observed in the vicinity of the five-fold axes (67 Å vs. 35 Å), and the thinnest capsid was observed at two-fold axes (36 Å vs. 25 Å). From the half sphere (Figure 3B, row B) and mid-section (row C) views of the particles, the elevation of the electron density could be observed at the outer tip of the five-fold channel, rendering a “hill” feature which was about 5–7 Å higher than the corresponding location on the empty VLP. The iso-surface maps of both types of VLP and their superimposition are shown in Figure 4 and Figure 5. It was rather apparent that the vertices of each VLP having a “tripod” feature (marked by letters a–c in Figure 4) appeared to rotate in a counter-clockwise direction in such a way that the a-pod turned downward into the capsid’s surface, and the c-pod moved upward, away from the surface planar. This twisting feature of a capsid protein has also been suggested in rhinovirus to allow the passage of RNA [10]. This rotation clearly increased the distance between the tip of each capsomere (known to be formed by a DE loop of anti-β-barrel strands), thus broadening the channel’s opening at the five-fold surrounding subunits (Figure 5, asterisk).

The inner surfaces of both VLPs were also strikingly different between the two VLP subclasses. Superimposition of the two VLPs demonstrated a marked increase in the interior capsid thickness (Figure 5). The thickness of the capsid interior (150–200 Å) of the dsDNA-filled VLP (depicted green in the mid-section, Figure 5D) occupied a much larger capsid area than that of the empty one. It should also be pointed out here that the capsid interior of the dsDNA-filled VLP showed a “bumpier” characteristic, with many nodular extensions that likely represent their focal interactions with dsDNA (Figure 5C,D, arrowheads). These nodes may be formed by clusters of positively charged amino acids found extensively in the C-terminus capsid sequence and known to be involved in interaction with nucleotides through ionic bonding [17]. It is, therefore, reasonable to postulate that these nodular capsid structures may represent feasible interaction points of the capsid interior surface with the IHHNV viral genome. However, whether these nodular structures resemble those of the natural virions remains to be elucidated.

## 4. Discussion

We have provided information on the ability of a small-sized VLP to encapsulate dsDNA designed from the IHHNV genome (3.9 kbp) into the IHHNV-VLP cavity, leading to a drastic capsid change. It must first be confessed that encapsulating dsDNA into the VLP’s cavity could only be partially controlled by our published capsid disassembly/reassembly method [16,18]. In this regard, approximately 30–40% of the VLPs were found to be fully loaded with the viral genome, leaving the other portion of particles empty or incomplete (Figure 2A). In addition, it should be noted that the packaging of dsDNA encountered different physical forces and packaging stress [19,20] compared to that of the natural, single-stranded (ss) DNA, form of the IHHNV genome. This might explain the low percentage of encapsulation of dsDNA. Alternatively, it also indicated that this VLP can accommodate the packaging of sizable dsDNA against a strong physical force. This flexibility of a small VLP to encapsulate dsDNA should be useful for specific applications, such as packaging and delivery of DNA vectors into the target cells and in the development of DNA vaccines against specific diseases.

Based on structural analysis and image reconstruction, the empty IHHNV-VLP generated in *E. coli* is similar in gross structure but somewhat different in fine structure compared to the empty IHHNV-VLP generated in *Sf9* (Figure 3, first and second columns) published earlier [2], despite being recombinantly produced in different host cells. The comparison of this empty VLP was then further extrapolated towards the dsDNA-filled VLP, where drastic alterations in the capsid structure were notable, particularly around the vicinity of the 5-fold axis (Figure 4 or 5). It has been reported that IHHNV has a capsid structure unique among all other known parvoviruses. Specifically, the P-domain on the surface of IHHNV is located around the five-fold area rather than at the three-fold area as in other known parvoviruses [2,21,22]. Apart from the P-domain, there is a fivefold channel/pore, which is formed by hydrophobic amino acids along the channel, and a glycine-rich motif at the tip of the channel, formed by the DE loop of the β-barrel motif. Packaging of dsDNA designed from a full-size viral genome into the VLP’s cavity consequently led to a counter-clockwise rotation and elevation of amino acid clusters around the five-fold axes (Figure 5). This structural alteration may reflect the widening of the five-fold channel vertex from a closed-type channel [2] to an open-type channel, the function of which may be related to either externalization of the capsid *N*-terminal peptide or packaging or release of the viral DNA [2].

Changes in capsid integrities of non-enveloped viruses have been reported to be dependent on at least three factors. The first factor is the length of the connecting loops in between the eight antiparallel β-barrel strands located within an individual capsomere [23]. The second factor is the dynamic interaction among subspecies of capsid proteins within the capsomere, which consequently renders structural changes in capsid architecture [5,8]. Nevertheless, these two factors may not be the case in the IHHNV for the following reasons: (1) there is no change in the length of the connecting loops during viral genome encapsulation (since they are the same type of particle as the empty particles), and (2) only a single type of capsid protein is present within the IHHNV capsomere. The third factor, where amino acid motifs within the capsid interior interact with the viral genome, would likely be the most favorable factor. In this regard, the dsDNA-filled reconstructed map and its analysis revealed the possible points of interaction between dsDNA loops and the corresponding amino acid motifs (Figure 3 and Figure 5), providing good support for this hypothesis. This type of interaction agrees well with previous evidence of capsid viral protein 1 (VP1) and the genome interaction reported in non-enveloped viruses. In simian virus 40 (SV40), capsid VP1 alone would rather self-assemble to form a tubular structure, whereas they form a viral icosahedral structure of about 40 nm in diameter in the presence of a viral genome [24,25]. In flock house virus (FHV), the length of packed RNA within the particle controls viral geometry [26], and the changes in RNA contents alter the dynamicity of the particles [27]. Together, we believe that packaging of dsDNA designed from the IHHNV viral genome generates a drastic change in capsid structure. It is important to note that the capsid alteration evident here is from an in vitro encapsulation study. Whether this structural change takes place during IHHNV assembly and maturation in host cells requires further extensive investigation.

## Data Availability

The data presented in this study are openly available in FigShare at https://doi.org/10.6084/m9.figshare.21507657.

## Supplementary Materials

The following supporting information can be downloaded at: https://www.mdpi.com/article/10.3390/v15010110/s1. Supplementary Figure S1: Protein profiles of IHHNV capsid protein during its purification process. The proteins were produced in *E. coli*, purified through nickel column chromatography, and the collected protein fractions were resolved by 12.5% SDS-PAGE and subjected to Coomassie blue staining. Supplementary Figure S2: Determination of the resolution for the reconstructed maps at the Fourier shell correlation (FSC) cutoff of 0.143. Note the resolution of the empty IHHNV-VLP is 10.2 A, which was constructed from 9394 boxed particles, and that of the dsDNA-filled VLP is 13.1 A, which was constructed from 3515 boxed particles.
