# Supplementary figures and images for: Viral Capsid Change upon Encapsulation of Double-Stranded DNA into an Infectious Hypodermal and Hematopoietic Necrosis Virus-like Particle
Source: Viruses. 2022 Dec 30;15(1):110. doi: 10.3390/v15010110 (PMC9867196; doi:10.3390/v15010110)

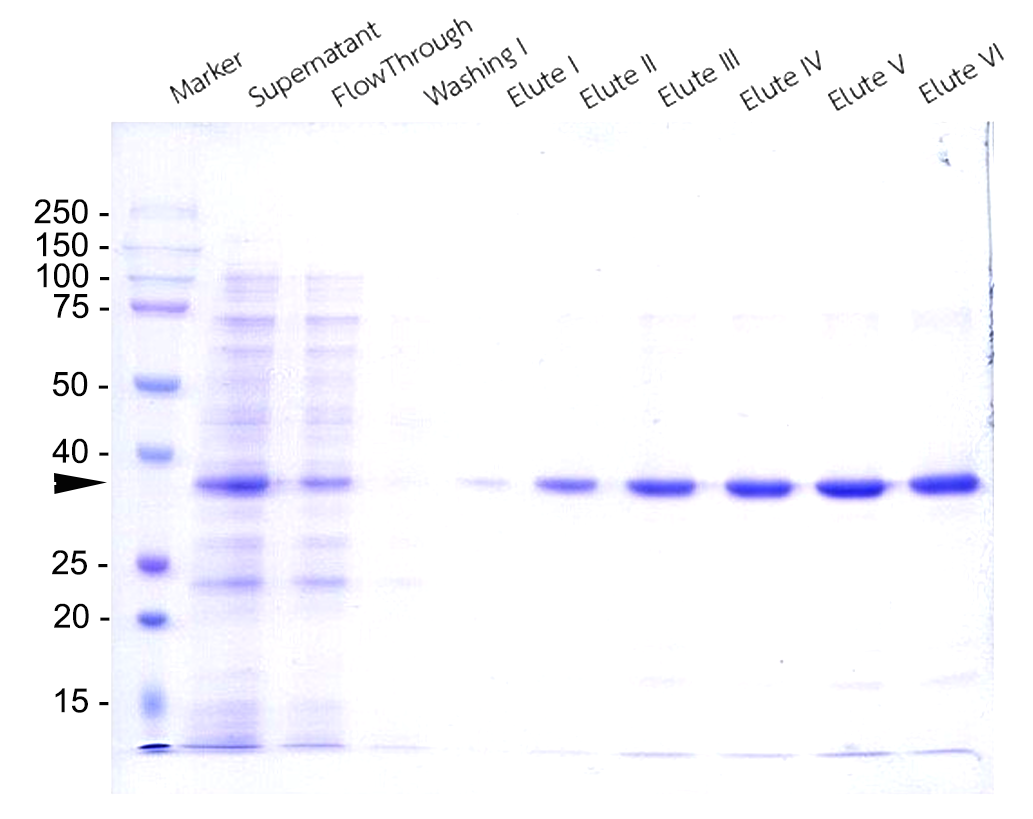

Supplement: Supplementary file 1 [file viruses-15-00110-s001.zip › viruses-2048393-supplementary Figure S1.tif]

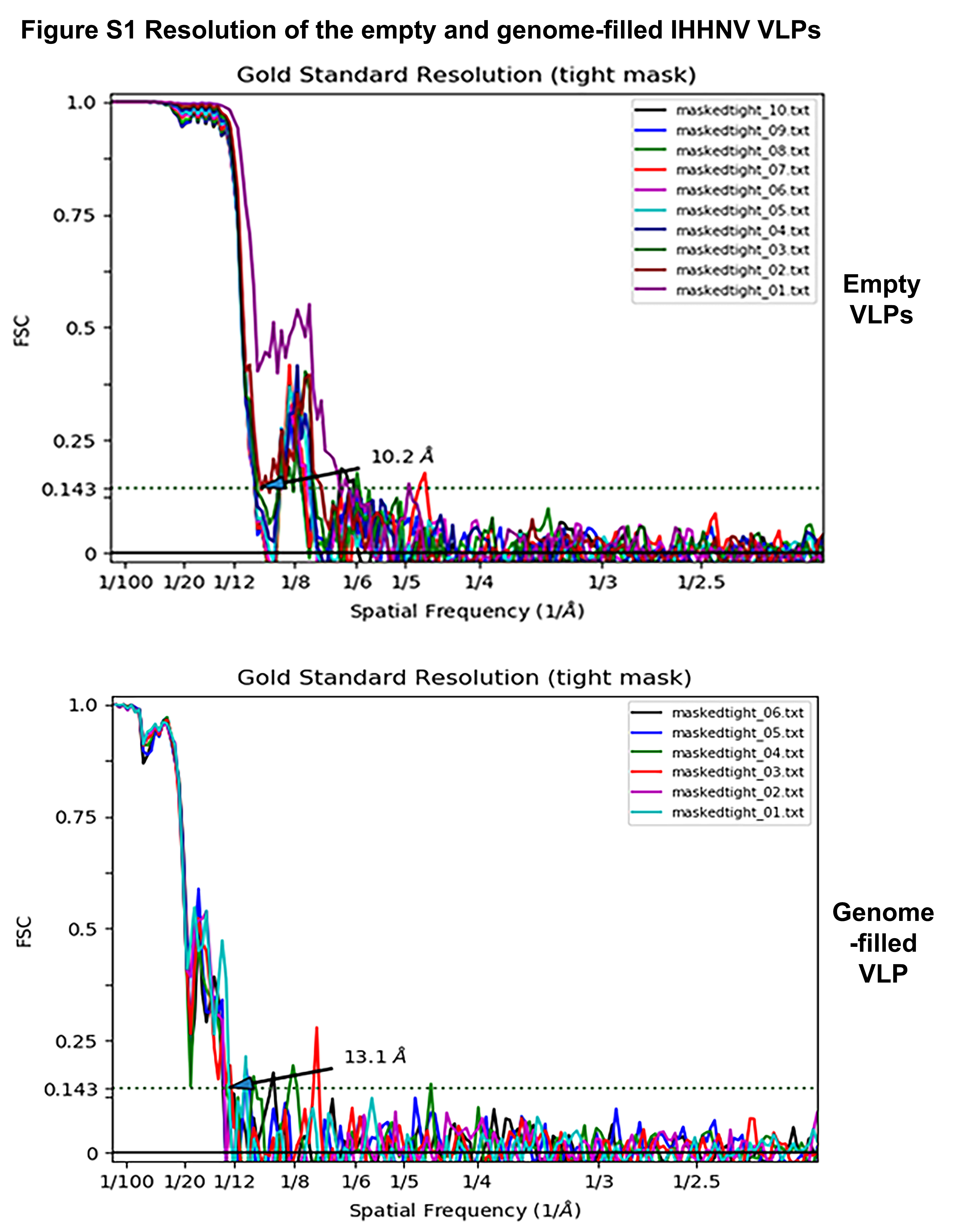

Supplement: Supplementary file 1 [file viruses-15-00110-s001.zip › viruses-2048393-supplementary Figure S2.tif]
